# Supplementary material for: Micronutrient Intake Adequacy in Men and Women with a Healthy Japanese Dietary Pattern
Source: Nutrients. 2019 Dec 18;12(1):6. doi: 10.3390/nu12010006 (PMC7019305; doi:10.3390/nu12010006)
Supplement: Supplementary file 1 [file nutrients-12-00006-s001.pdf]

Table 1. Factor loading matrix for the healthy Japanese dietary pattern identified by principal component analysis.

Food	Factor loading [a]
Carrots/pumpkin	0.697
Green leaves vegetable	0.692
Other root vegetables	0.689
Cabbage/Chinese cabbage	0.652
Japanese radish/turnip	0.652
Mushrooms	0.640
Seaweeds	0.558
Tofu/atsuage [b]	0.483
Lettuces/cabbage (raw)	0.433
Tomatoes	0.403
Potatoes	0.373
Other fruit	0.358
Lean fish	0.331
Small fish with bone	0.321
Persimmons/strawberries/kiwifruit	0.314
Oily fish	0.307
Citrus fruit	0.261
Natto [c]	0.245
Dried fish/salted fish	0.244
Egg	0.233
Pickled green leaves vegetable	0.205
Green tea	0.184
Squid/octopus/shrimps/shellfish	0.181
Pickled other vegetables	0.179
Chicken	0.174
Canned tuna	0.153
Mayonnaise/dressing	—
Japanese confectioneries	—
Milk/yogurt	—
Law fat milk	—
Pork/beef	—
Black tea/oolong tea	—
Hum/sausage/bacon	—
Western-type confectioneries	—
Liver	—
Coffee	—
100% fruit and vegetable juice	—
Ice cream	—
Bread	—
Rice crackers/rice cake/okonomiyaki [d]	—
Japanese noodles	—
Sake	—
Wine	—
Miso soup	—
Buckwheat noodles	—
Cola drink/soft drink	-0.161
Whisky	-0.170

Pasta	-0.171
Shochu	-0.206
Beer	-0.300
Chinese noodle	-0.322
Rice	-0.333
Variance explained (%)	10.2

[a] A factor loading less than ± 0.15 is represented by a dash for simplicity; [b] Deep-fried tofu; [c] Fermented soybeans; [d] Savory pancake with various ingredients (meat, fish, and vegetable).
